# Global Invasion of *Lantana camara*: Has the Climatic Niche Been Conserved across Continents?

**DOI:** 10.1371/journal.pone.0111468

**Published:** 2014-10-24

**Authors:** Estefany Goncalves, Ileana Herrera, Milén Duarte, Ramiro O. Bustamante, Margarita Lampo, Grisel Velásquez, Gyan P. Sharma, Shaenandhoa García-Rangel

**Affiliations:** 1 Centro de Ecología, Instituto Venezolano de Investigaciones Científicas, Caracas, Venezuela; 2 Departamento de Estudios Ambientales, Universidad Simón Bolívar, Caracas, Venezuela; 3 Departamento Cs. Ecológicas, Facultad de Ciencias, Universidad de Chile, Santiago, Chile; 4 Instituto de Ecología y Biodiversidad, Facultad de Ciencias, Universidad de Chile, Santiago, Chile; 5 Department of Environmental Studies, University of Delhi, Delhi, India; Instituto de Agricultura Sostenible (CSIC), Spain

## Abstract

*Lantana camara*, a native plant from tropical America, is considered one of the most harmful invasive species worldwide. Several studies have identified potentially invasible areas under scenarios of global change, on the assumption that niche is conserved during the invasion process. Recent studies, however, suggest that many invasive plants do not conserve their niches. Using Principal Components Analyses (PCA), we tested the hypothesis of niche conservatism for *L. camara* by comparing its native niche in South America with its expressed niche in Africa, Australia and India. Using MaxEnt, the estimated niche for the native region was projected onto each invaded region to generate potential distributions there. Our results demonstrate that while *L. camara* occupied subsets of its original native niche in Africa and Australia, in India its niche shifted significantly. There, 34% of the occurrences were detected in warmer habitats nonexistent in its native range. The estimated niche for India was also projected onto Africa and Australia to identify other vulnerable areas predicted from the observed niche shift detected in India. As a result, new potentially invasible areas were identified in central Africa and southern Australia. Our findings do not support the hypothesis of niche conservatism for the invasion of *L. camara*. The mechanisms that allow this species to expand its niche need to be investigated in order to improve our capacity to predict long-term geographic changes in the face of global climatic changes.

## Introduction

The West Indian Lantana, *Lantana camara* L., is considered among the most harmful invasive species in the world [1], [2]. Its ability to form dense monospecific stands through the high reproductive capacity and allelopatic exclusion of other plant species can significantly reduce the productivity of agricultural systems and negatively impact the biodiversity of the invaded regions [3]. Although its natural range extends from Mexico to Brazil, the species has established populations in more than 60 countries worldwide [4] causing large economic losses in some of these. For example, in Australia losses associated with the introduction and expansion of this weed has been estimated in the order of $2.2 million per annum [5].

Mechanical and chemical management are currently used for the eradication and control of *L. camara*. These options, however, are often costly and inefficient because invaded areas tend to be vast with limited access [6], [7]. On the other hand, biological control agents seem insufficient for reducing the abundance of *L. camara* to manageable levels (*e.g.* [8]). Thus, as with many invasive plant species, prevention is the first recommendation to limit the expansion of *L. camara*. The efficient implementation of preventive actions to stop the arrival and establishment of invasive species relies on the correct identification of potentially suitable areas.

Species distribution models (SDMs) are powerful tools for predicting the potential distribution of invasive plants (*e.g.* [9]–[11]). Based on spatial correlations between species occurrence and environmental variables, SDMs identify sets of variables associated with the presence of invasive species to project their requirements onto the geographic space [12]–[14]. One fundamental assumption underlying SDMs is the principle of niche conservatism, which states that species tend to preserve their ancestral niches requirements over time and space [15]–[18]. In the context of biological invasions, a niche is conserved whenever the species occupies the same environmental conditions in its native and invaded ranges [14], [18]. If, on the contrary, the environmental conditions where the species occurs differ between the native and the invaded ranges, the species' niche is considered to have shifted. Recent studies have suggested that niche conservatism does not occur in all invasive species [19]–[21]. Based on a comprehensive study including 50 invasive plants species, Petitpierre *et al.* [21] found that 15% of the invasive plants species evaluated did not conserve their niche during invasion. Although this represents a small proportion of all invasive species, it highlights the need to test the niche conservatism assumption before using SDMs. When the assumption of niche conservatism fails, SDMs underestimate the potential distribution of invasive plants. Alternative hypotheses must be formulated to account for the observed niche shift and new methods are required to increase predictive value of these models [22].

Several authors have predicted current and future distributions of *L. camara*, under possible scenarios of global change [23]–[26]. Some foresee a contraction of its distribution at global scale [24], while others expect expansion in some particular regions (*e.g.* Australia and China) [25], [26]. All of these studies assume that the niche of this species has been conserved, although this premise has not been tested. Some characteristics of *L. camara* and its invasion history could favor niche shifts due to adaptive evolution. First, the species complex history of introductions (see [27]) has probably involved several genetic bottlenecks. Second, its polyploid condition and ability to hybridize are characteristics that promote rapid evolutionary changes [28], [29]. Third, more than 600 ornamental varieties have been produced as a result of artificial selection and hybridization [6], [29]. It is now widely recognized that naturalized populations of *L. camara* are morphologically different than populations in its native range [30]. Therefore, it is possible for naturalized populations to differ genetically from the original ancestral populations, to the extent that their niches are no longer exact.

Here, we tested the niche conservatism hypothesis for *L. camara* using a niche dynamics analysis [21], [31]. Estimated niches were projected onto the geographic space to identify potentially suitable areas for *L. camara,* and to evaluate the possible implications of a niche shift on its potential distribution in three invaded regions, Australia, Africa and India (*e.g.* [19]).

## Materials and Methods

### Study species

*Lantana camara* (Verbenaceae, a perennial evergreen shrub native to the Neotropics, was introduced into Europe from Brazil as an ornamental plant in the 17^th^ century [32]. For the next 100 years after its initial introduction, this species was extensively exported from Europe to Africa, Asia, America and Oceania. Although it established populations in several countries, *L. camara* only became invasive throughout tropical, subtropical and warm temperate areas [33]. This species currently occupies millions of hectares in South Africa, Australia and India, and continues to expand (reviewed by [27]). *L. camara* has several traits that explain its high invasiveness: it is autocompatible; it is pollinated by different groups of insects (*e.g.* butterflies and honey bees); it has a high seed output with birds dispersing the seeds over long distance; it forms large seedbanks and has high potential of vegetative reproduction; it is a fire-tolerant and it has a high phenotypic plasticity. Also, this species frequently outcompetes native flora (for review see [3]).

Despite its cosmopolitan distribution, the taxonomic status of *L. camara* has not been resolved yet. It is considered a species complex, *L. camara sensu lato*, consisting of several taxa that are morphologically and ecologically very similar to the initial description of the species [29]. For the purpose of this study, *L. camara sensu lato* will be hereafter referred as *L. camara*.

### Occurrence data

**Figure 1:**
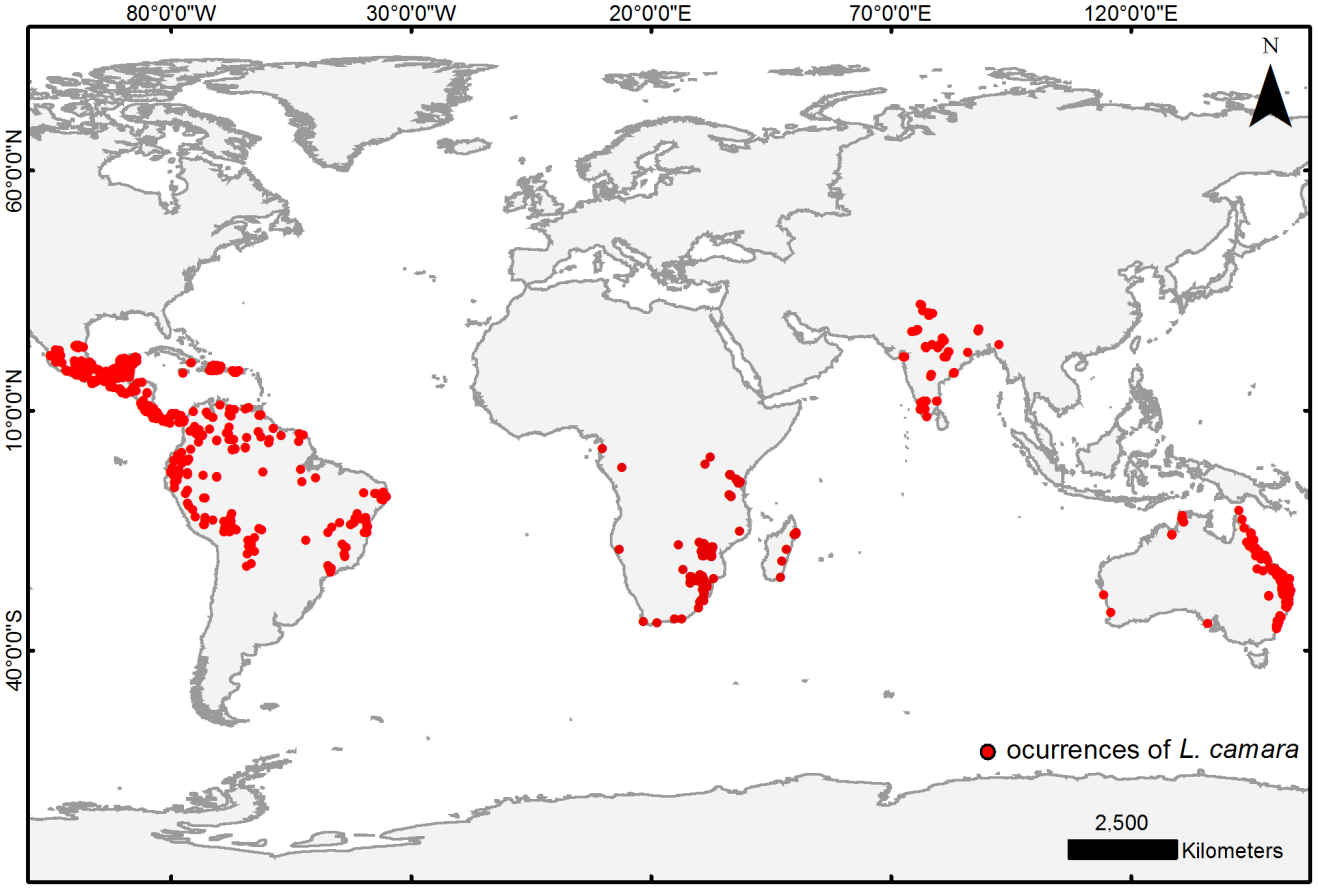
Filtered occurrences of *L. camara* used for this study.

The occurrences of *L. camara* from their native and introduced ranges were obtained from several sources (Table 1). To filter occurrence data, we selected records collected after to 1950 that included a detailed description of the locality. A total of 896 occurrences were used: 167 from Australia, 96 from Africa, 84 from India and 549 from its native range in America (Figure 1; Table S1). We defined its native range as the geographic area between 24°N (Mexico) and 24°S (Southern Brazil), and constrained our analysis to Australia, Africa and India, where the species has invaded and caused major impact [27].

**Table 1:** Biodiversity databases used to obtain occurrences of *L. camara*.

Database	Description
GBIF	Global Biodiversity Information Facility: Network for the exchange of biodiversity data through the internet. Compiles data from specimens deposited in museums, herbaria, and published in atlases and references worldwide. Web page:http://data.gbif.org/welcome.htm
REMIB	“Red Mundial de Información sobre Biodiversidad”: It is an interagency network that shares biological information. Itconsists of research centers nodes that host the digitized scientific collections. Web page: http://www.conabio.gob.mx/remib_ingles/doctos/remibnodosdb.html
TROPICOS	TROPICOS is a Botanical Information System. It's a public-access botanical database with records of millions of specimens, images and references by the Department of Bioinformatics of the Missouri Botanical Garden. Here we used the”Geographical Search” tool.Web page: http://www.tropicos.org/SpecimenGeoSearch.aspx
PRECIS	Information Computerized Pretoria: This is a digital database on the state of biodiversity in southern Africa by the South AfricanBiodiversity Institute (SANBI). It includes data from herbaria and published. Web page:
Zimbabwe	Data collection of scientific research on plants in Zimbabwe. The data are collected and compiled by the authors of the website:Hyde, M.A., Wursten, B.T. & Ballings, P.Web page: http://www.zimbabweflora.co.zw/
AVH	Australia’s Virtual Herbarium: It is a compilation of occurrence data and specimens from several herbaria in Australia. Webpage : http://chah.gov.au/avh/avhServlet?task=simpleQuery

### Environmental data

We chose 12 from the 20 environmental variables available in the WorldClim dataset [34]. This selection was based on natural history data of *L. camara* [3] and the contribution of these variables to a previous test model. The remaining eight variables were not included because they had no discriminatory power (low contribution); for example, variables for which the species showed wide tolerance (*i.e.* altitude) or those with most values outside the physiological tolerance of *L. camara* (*i.e.* minimum temperature of coldest month). Also, to minimize redundancy due to potential multicollinearity among variables, we omitted highly correlated variables. Using a cross-correlation analysis in software *R* [35], we estimated the Pearson's correlation coefficient between pairs of variables, and when >0.9, we selected from the pair that variable more relevant for the ecology of the species. Thus, the initial set of 12 variables was further reduced to six: i) annual mean temperature (BIO1), ii) maximum temperature of the warmest month (BIO5), iii) annual precipitation (BIO12), iv) precipitation of the driest month (BIO14), v) precipitation seasonality (BIO15) and, vi) precipitation of the warmest quarter (BIO18). Raster layers (resolution = 2.5 arc-min∼25 km^2^) for these climatic variables were obtained from WorldClim dataset (http://www.worldclim.org/) [34].

### Niche analysis

We used PCA-env analyses [31] to assess the similarity between niches. This procedure allowed us to evaluate the hypothesis of niche conservatism between native and invaded ranges. Three PCA were conducted to compare the native niche of *L. camara* with its niches estimated for each invaded region: Australia, Africa and India. For each PCA, we used the first two axes to define the environmental space. The environmental space was divided into 100×100 cells, and the occurrence points were converted into densities of occurrences, *o_ij_*_,_ using a kernel smoothing function. Then, 10,000 randomly generated points (*i.e.* pseudo-absences) were used to estimate the density of available environments, *e_ij_*, in each cell of the environmental space. Based on the values of *o*_ij_ and *e*_ij_ an occupancy index, *z_ij_*, was estimated. This metric allowed for the unbiased comparison of occurrence densities, when environments were not equally available. Finally, the values of *z_ij_* were plotted on the environmental space to delimit the climatic niche occupied by *L. camara* in their native and invaded ranges.

We used four approximations to compare invaded niches with native niche [36]: (i) qualitatively changes in the niche center, cells with the highest values of *z_ij_*; (ii) niche overlap *D*; (iii) niche equivalence, the correspondence between an observed *D* and that expected by randomly reallocating the occurrences from both entities on both ranges, and (iv) the niche similarity, the comparison between an observed *D* and that expected by randomly reallocating the occurrences in only one of the ranges. For the equivalence test, the null hypothesis (*i.e.* niches are not identical) is rejected if *p*<0.05. For the similarity test, in contrast, a *p* value>0.05 indicated that niches were no more similar than expected by chance.

Additionally, we indentified niche zones within the environmental space by overlapping the native and invasive niches according to Petitpierre *et al*. [21]: (i) unfilled (*U*), the zone on the native niche not shared with the invaded niche; (ii) overlap (*O*), the zone shared between native and invasive niches; and (iii) expansion (*E*), the zone on the invaded niche not shared with the native niche. While the *O* values measured the proportion of niche conserved, the *E* values estimated the proportion niche expanded. The unfilled zone (*U*) assesses the fraction of niche not yet occupied by the species in the invaded range.

### Species distribution models

We used species distribution models (SDMs) to predict potential suitable areas for the invasion of *L. camara*. One limitation of these models is that they do not distinguish if a particular occurrence is associated with a high abundance (source) or a low abundance population (sink). Therefore, an area classified as “suitable” corresponds to an area with high establishment risk of the species, but not necessarily with a high invasion risk [37]. However, for exotic plants with high invasive potential, as *L. camara,* the risk of establishment can be considered equivalent to of the risk of invasion.

We generated potential distributions of *L. camara* in their native and invaded regions using MaxEnt (*v*. 3.3.3.k) [38]. Using a maximal entropy function, this software estimates the probability of occurrence based on the environmental characteristics of the habitats where the species is present [38]. We choose use the MaxEnt’s default settings after contrasting several models, and corroborating that this selection gave the best model based on the Akaike information criterion (AIC). For each study region, presence data was used to construct a set of 10 candidate models. Then, we selected the best four models (one for each region) based in the AIC, to generate potential distributions of *L. camara* in its native region (native-to-native distributions), and in each of the invaded regions (invaded-to-invaded distributions). We then projected the model for the native region onto each invaded region to generate three additional native-to-invaded distributions (native-to-Africa, native-to-Australia, and native-to-India). Whenever the niche analyses indicated expansion (*E*>0) or niche shift (niche similarity<expected), we projected this modified niche onto the rest of the invaded regions (modified-niche-to-invaded). The latter illustrates the effect of possible niche changes in the potential distribution of *L. camara* in the invaded regions. All potential distributions were determined using a threshold value equivalent to the 10^th^ percentile of the probability of occurrence. We overlaid the predicted native-to-invaded distributions with the predicted invaded-to-invaded distributions to estimate the unfilled area (*U*), the overlap area (*O*), and the expansion area (*E*). This similar procedure was used to overlay the modified-niche- to-invaded distribution with the invaded-to-invaded distribution [21].

### Model evaluation and validation

To evaluate model accuracy we used a cross-validation method. For each region, 90% of the occurrence points were set as training data, and the remaining 10% as test data. To assess the model’s accuracy in predicting the species’ presence in a particular grid cell, we used the area under the curve (AUC) of the receiver operating characteristic (ROC) estimated by MaxEnt for the training and test data sets. Using an R package developed by B. Petitpierre, we also estimated the Boyce Index, *i.e.* threshold-independent accuracy estimator based on the Spearman rank-correlation coefficient between the predicted points and the predicted areas for both data sets. For latter analysis, we randomly selected 10,000 pixels for each model prediction to reduce computational time. The omission rates of occurrences in each invaded region were also calculated to assess how accurately the native-to invaded distributions predicted the occurrence points on the invaded regions.

### Climatic analogy between native and invaded ranges

To assess the risk of extrapolating species distributions to regions with substantially different climates [39], we evaluated the climatic analogy between native and invaded regions using the Multivariate Environmental Similarity Surface (MESS) analysis in MaxEnt [40]. We identified the “most dissimilar” climatic variable and its geographic location, and then we compared the values of these climatic variables for the non-analogous regions by a *Student-t* test.

## Results

### Niche analysis

**Figure 2:**
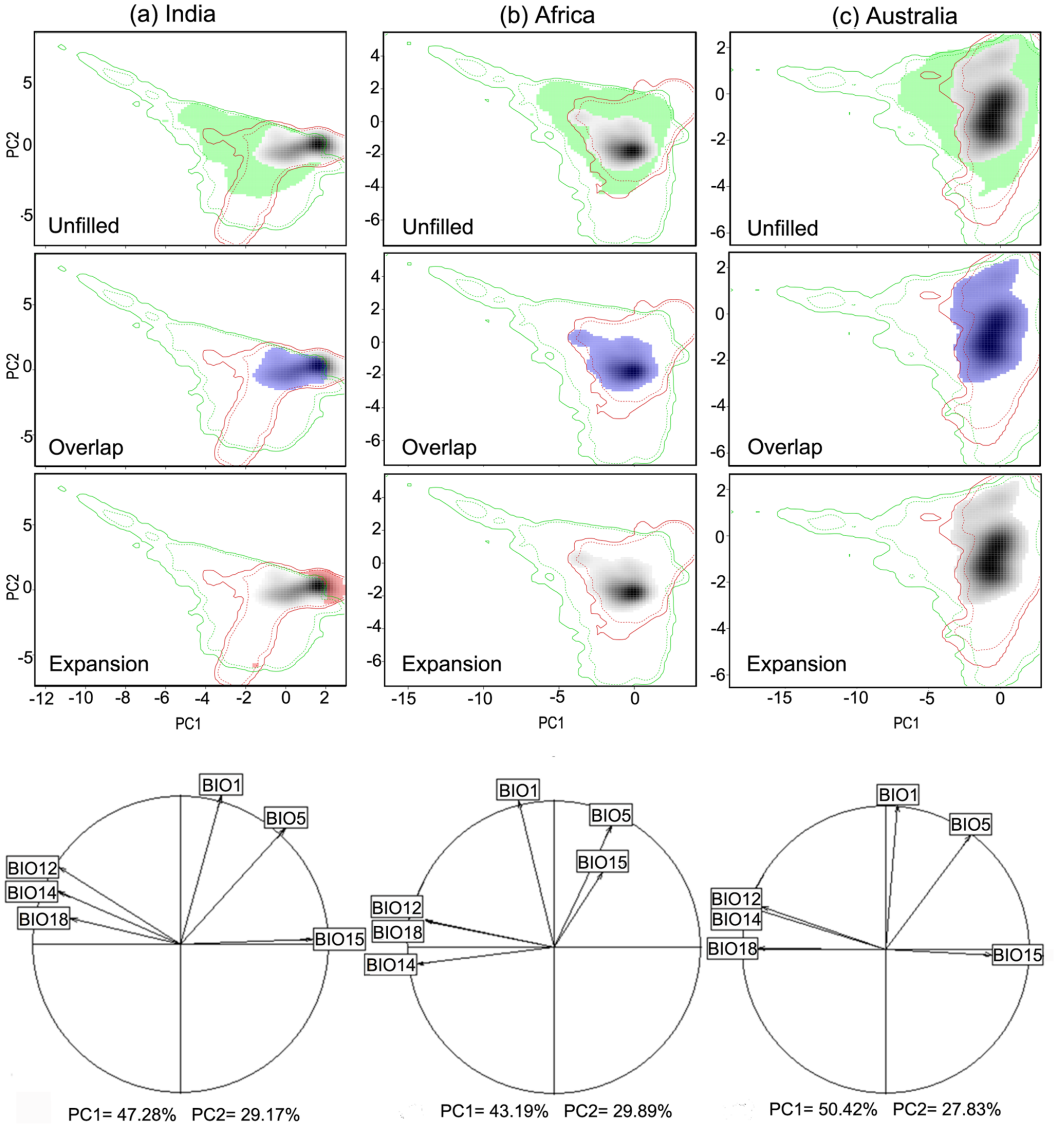
Niche dynamics of *Lantana camara:* from native to invaded ranges. The contour lines delineate the available niche in its native range (green) and in its invaded range (red) in India (column a), Australia (column b) and Africa (column c). The solid and dashed contour lines illustrate, respectively, 100% and 50% of the available (background) environment. The colored areas correspond to the unfilled zone (green; line 1), the overlap zone (blue; line 2), or the expansion zone (red; line 3) resulting from overlaying the native niche with the invaded niche. The last line shows the correlation circles, which indicate the weight of each bioclimatic variable on the niche space defined by the first two principal component axes. The predictor climatic variables are BIO1 (annual mean temperature), BIO5 (temperature of warmest month), BIO12 (annual precipitation), BIO14 (precipitation of driest month), BIO15 (precipitation seasonality), BIO18 (precipitation of warmest quarter).

The PCA-env analyses of native and invaded regions showed changes in the size of the niches and in the position of the areas with the highest values of *z_ij_* (*i.e.* highest density of occurrences) (Figure 2). In all cases, the native region had a greater niche breadth than any of the invaded regions. In India, the shift on the highest density of occurrences surpasses the limits of the native niche (Figure 2 a), while in Africa and Australia, this shift occurs within these limits (Figures 2 b and c, respectively). In the correlation circle, the arrow directions show that, in India, the niche moved towards lower values of precipitation (BIO18), and greater seasonality of the latter (BIO 15) (Figure 2 a). In Africa towards colder climates (BIO1) (Figure 2 b), and in Australia, the niche moved towards lower temperatures (BIO1) (Figure 2 c). These plots also identified presence of unfilled niches in all invaded regions and niche expansion only in India, where the ∼20% of climatic conditions occupied in India are not available in the native region (Table 2; Figure 2).

**Table 2:** Niche dynamics values estimated using climatic conditions in the native and invaded regions.

Niche comparisons	Overlap (*O*)	Unfilled (*U*)	Expansion (*E*)
Native *vs.* Australia	1	0.17	0
Native *vs.* Africa	1	0.47	0
Native *vs.* India	0.79	0.23	0.20

**Figure 3:**
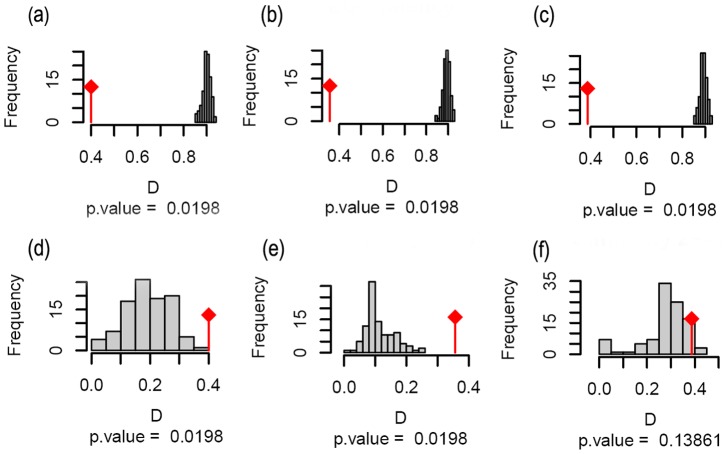
Statistical tests for niche comparisons between native and invaded regions. Observed frequencies for the niche overlap index (*D*) in relation to the expected *D* for *p* = 0.05. The first line shows the tests for niche equivalency (a, b and c) and the second line for niche similarity (d, e, and f). The first column compares niches between the native range and Australia (a and d), the second column between the native range and Africa (b and e) and the third column between the native range and India (c and f).

The niche equivalency tests confirmed that niches from the three invaded regions are not identical to the native niche (Figure 3 a–c). The niche similarity tests showed that the niches of *L. camara* in Australia and Africa are more similar to the niche of the native region than would be expected by chance (Australia: *D* = 0.3, *p* = 0.02; Africa: *D* = 0.4, *p* = 0.02; Figure 3 d–e). In Australia and Africa, *L. camara* only occupies areas with similar climatic condition to those found in its native range. In contrast, the climate niches in India and the native region are not more similar than expected by chance (*D* = 0.3, *p* = 0.4, Figure 3 f). This indicates that in India the occupation of the species does not follow a pattern expected by native niche requirements and seems to be random. In India, *L. camara* occupies various climatic conditions, some of which are similar to the climatic condition in its native range, while others are different.

### Species distribution models

The selected model showed proper fit of the data. The AUC and the continuous Boyce index were high for the training or test data sets (Table 3). The projection of native model onto all invaded regions had an omission rate of 10% of occurrence points, indicating an adequate prediction. Most of these omissions occurred in India. Similarly, the model training with the Indian range and their extrapolation to Australia and Africa had a low omission rate (14%) (data not shown). These results suggest that both models are informative and did fairly well predictions.

**Table 3:** Evaluation index values (AUC and Boyce Index) and omission rate for the obtained models.

Model	AUC_training_	AUC_test_	B_training_	B_test_
Native	0.811	0.717	0.998	0.899
Australia	0.976	0.944	0.977	0.669
Africa	0.968	0.962	0.934	0.576
India	0.823	0.802	0.959	0.610

Values for training and test evaluation are shown.

**Figure 4:**
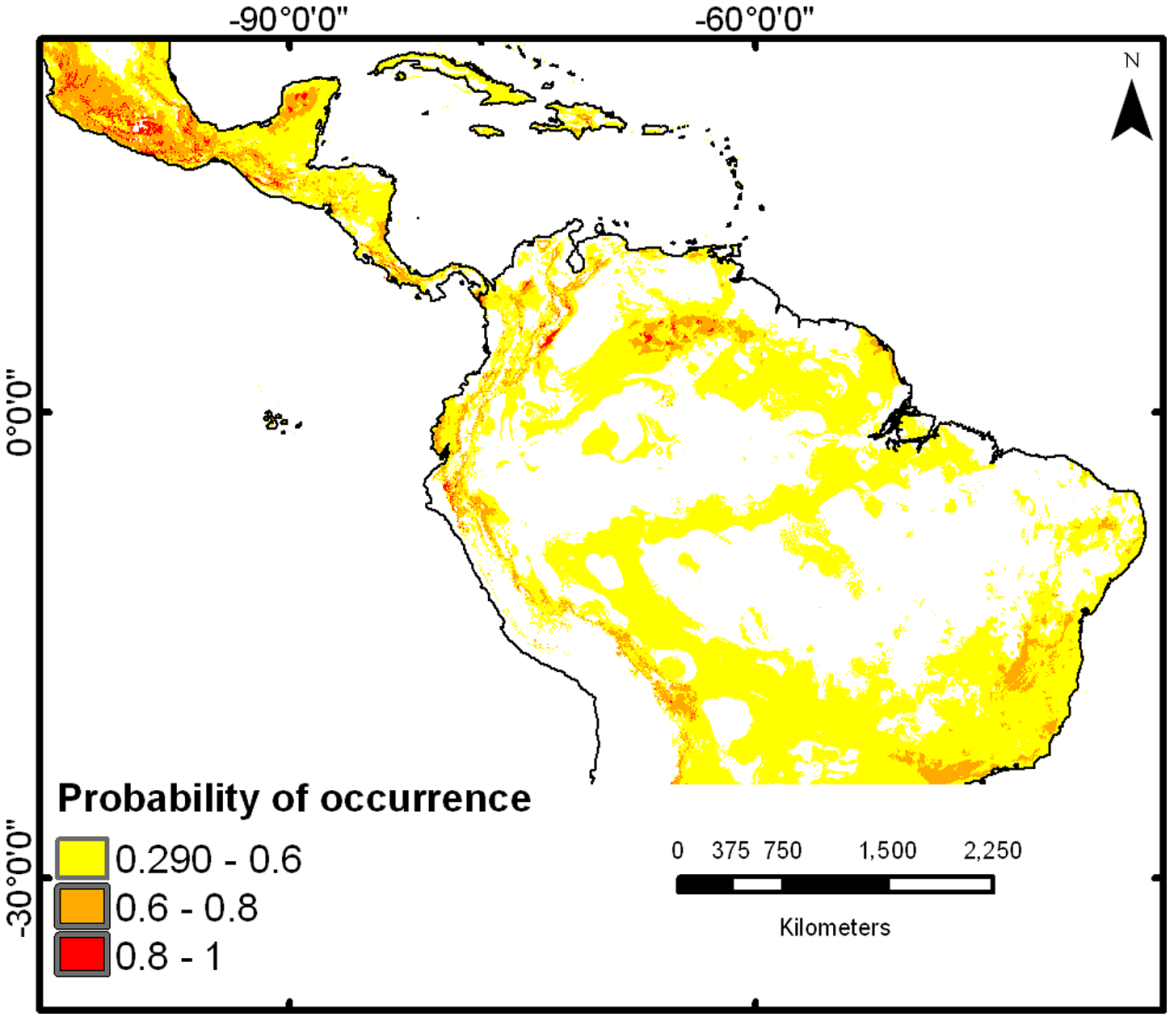
Potential geographic distribution of *L. camara* in its native region. Predictions are based on current occurrences in the Neotropics and the climatic data from the places where this plant inhabits there.

**Figure 5:**
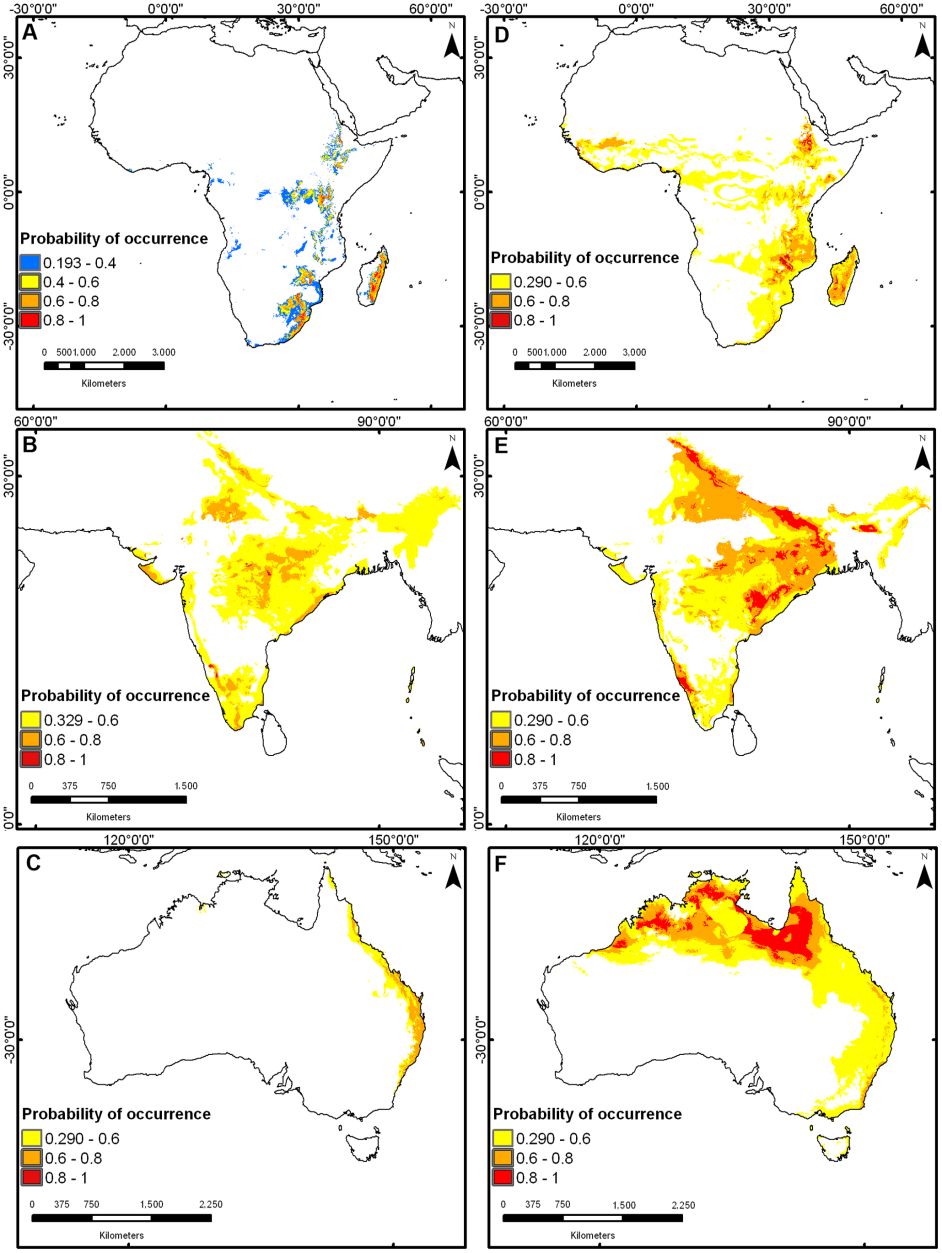
Potential geographic distributions of *L. camara* in each invaded region. The first line shows the predicted geographic distributions in Africa (a, d), the second line in India (b, e) and the third in Australia (c, f). The first column illustrates predictions based on invaded-to-invaded projections (a, b, c), and the second column based on native-to-invaded projections (d, e, f).

The native potential distribution showed the highest probabilities of presence across southwest of Mexico, the lower slopes of the Andean cordillera in Colombia, and some savannas and evergreen forests in southern Venezuela – all characterized by dry and warm climates – showed the highest presence probabilities (Figure 4). In general, native-to-invade models generated wider distributions than invade-to-invade models (Figure 5 a–f). In the invadedtoinvaded distribution the higher probability of presence occurred in Cape Town, South Africa, center of India and all of the east coast of Australia (Figure 5 a–c). In the native-to-invaded distribution (Figure 5 d–f), the higher probability of presence occurred in Madagascar, around the Malawi Lake in Africa, in northeast India and northern Australia. No climatically suitable areas for *L. camara* were identified in central or southern Australia.

**Figure 6:**
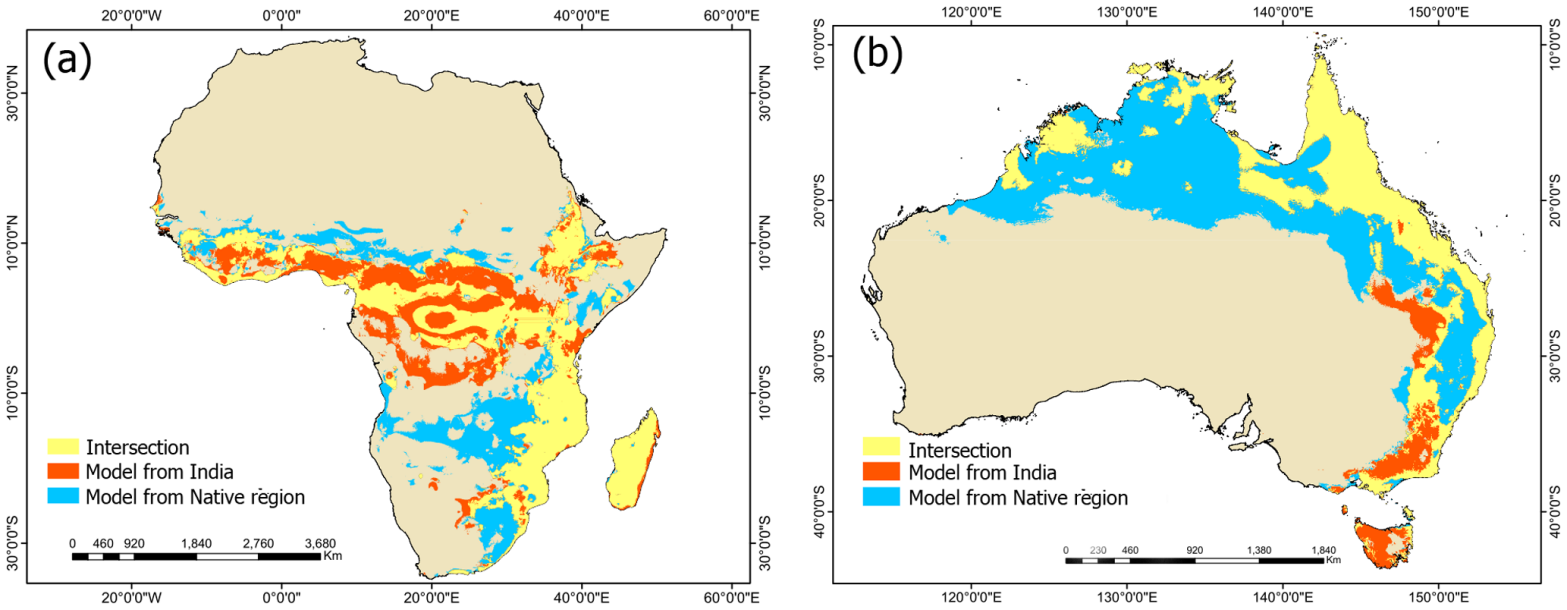
New potentially invadable areas resulting from the observed niche shift in India. Overlay of the potential geographic distributions in Africa (a) and Australia (b) estimated from the native niche and the modified niche in India. The orange areas identify new vulnerable areas. They correspond to locations predicted as unsuitable according to its current distribution in its native range, but as suitable based on its current distribution in India.

Australia and Africa were the regions with the highest unfilled areas; more than 70% of the predicted distribution for *L. camara* in both regions are not occupied yet (Table 4). India, on the contrary, was the region with the highest value of overlap area; 68.3% of the predicted distribution is occupied by the species. Expansion areas were small for Australia and Africa (<3%). Only in India *L. camara* occupied areas not predicted by its distribution in the native range (Table 4). When the India model was projected onto Australia or Africa (modified-niche-to-invaded), the unfilled area in both regions (*i.e*. new potential distribution areas) increased in 6.1% in Australia and 24.3% in Africa (Table 5; Figure 6).

**Table 4:** Percent overlap, unfilled and expansion areas in Australia, Africa and India obtained by overlaying the native-to-invaded distributions with the invaded-to-invaded distributions in each invaded region.

Distribution comparisons	Overlap area	Unfilled area	Expansion area
Native *vs.* Australia	10.6% (∼3.0×10^5^ km^2^)	89.2% (∼2.8×10^6^ km^2^)	0.2% (∼5.3×10^3^ km^2^)
Native *vs.* Africa	26.8% (∼1.9×10^6^ km^2^)	70.2% (∼6.8×10^6^ km^2^)	3.0% (∼2.1×10^5^ km^2^)
Native *vs.* India	68.3% (∼8.8×10^5^ km^2^)	19.8% (∼1.9×10^6^ km^2^)	11.9% (∼2.6×10^5^ km^2^)

Percentages were adjusted by the predicted total area for *L. camara* in each invaded region.

**Table 5:** Percent unfilled and expansion areas in Australia and Africa obtained by overlaying the modified-niche-to-invaded distributions with the invaded-to-invaded distributions in both invaded region.

Distribution comparisons	Unfilled area	Expansion area
Expanded *vs.* Australia	90.0% (∼2.7×10^6^ km^2^)	6.1% (∼1.7×10^5^ km^2^)
Expanded *vs.* Africa	78.7% (∼7.7×10^6^ km^2^)	24.3% (∼2.4×10^6^ km^2^)

Percentages were adjusted by the predicted total area for *L. camara* in both invaded regions.

### Climatic analogy between native and invaded ranges

**Figure 7:**
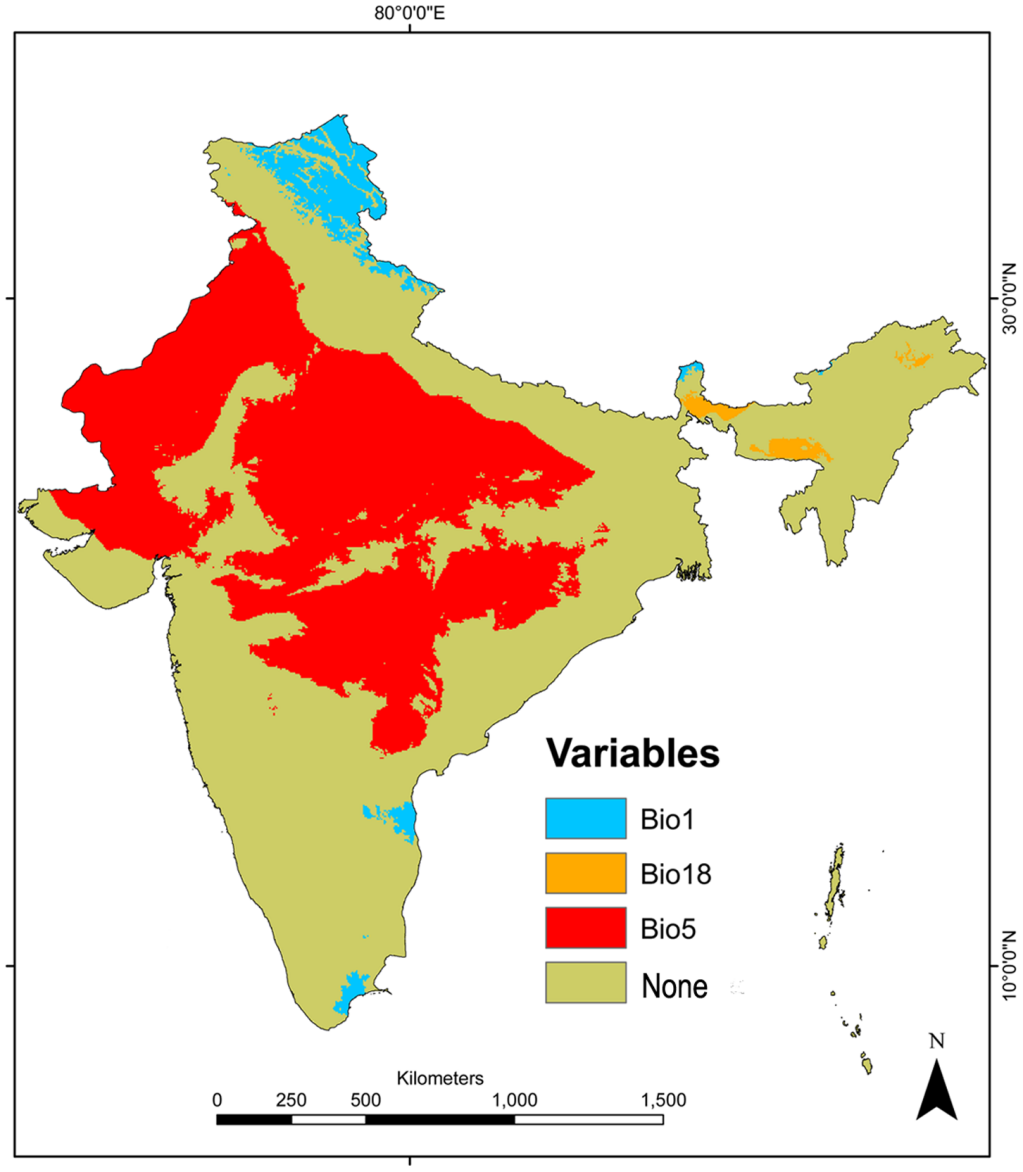
Climatic analogy between the native range of *L. camara* and its invaded range in India. Using the multivariate environmental similarity surface (MESS) we identified BIO1 (annual mean temperature), BIO18 (precipitation of the warmest quarter) and BIO5 (temperature of the warmest month) as the most dissimilar variables. The red, blue and yellow areas identify locations in India with values for BIO5, BIO1 and BIO18, respectively, outside the observed value range in its native region.

**Figure 8:**
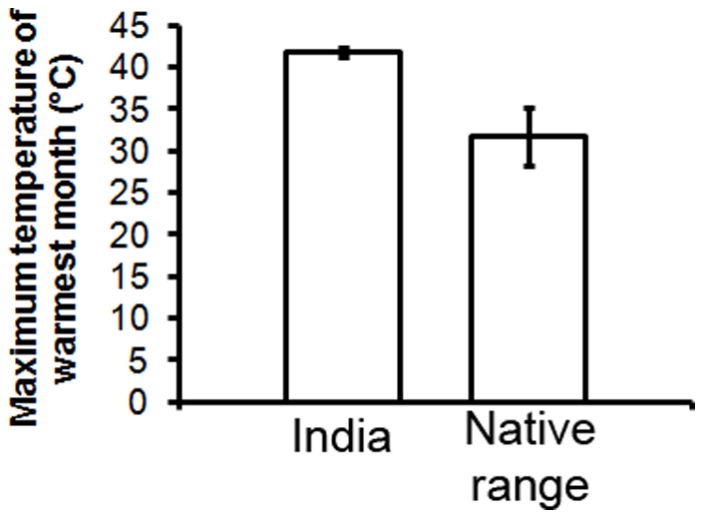
Differences in maximum temperature of the warmest month between the native range of *L. camara* and its invaded range in India. The mean values and their standard errors were estimated using the temperature of the warmest month at each location where this plant is present in India and in its native range.

According to the MESS analysis, India was the only region with climatic conditions non-analogous to those observed in the native region (Figure 7). In India, 34% of the occurrences are in locations where maximum temperatures of the warmest month (BIO 5) reach in average 43°C, a value that is significantly higher (*t*-test, *t* = 87.159, *p*<0.001, see Figure 8) than the average of maximum temperature of the warmest month in the native region (35°C).

## Discussion

For the first time the hypothesis of niche conservatism is evaluated for the *L. camara* invasion. Our results demonstrate that even though the niches occupied by *L. camara* in Africa and Australia are subsets of its native niche in the Neotropics, in India this species’ niche shifted significantly towards warmer climates, with temperatures that frequently exceed the maxima recorded in its native region. The presence of *L. camara* in novel climatic conditions indicates that its niche has not been conserved throughout the process of invasion, therefore suggesting a greater capacity to invade new regions than previously thought.

Niche shift has been documented in several invasive plant species [20], [21]. In Australia, a continent where biological invasions are common, 19 invasive plant species are known to have shifted into novel biomes not present in their native range [20]. In theory, niche shifts may conceal one of two mechanisms. First, the species could find new suitable conditions in the invaded regions that are absent from their native range (*i.e.* non-analog climate) but lie within their tolerance ranges or fundamental niche [41]. This mechanism involves a shift only in the realized niche (*i.e.* filling a pre-adapted niche) and whether it should be considered as a true niche shift is still controversial [21], [41]. Secondly, the species can undergo genetic changes that allow it to adapt to conditions outside their tolerance ranges, changing its fundamental niche [42]. Identifying the underlying mechanism using SDMs is difficult, and has prompted a recent discussion on whether these analyses should be constrained to niche shifts between analog climates exclusively [21], or include non-analog climates [41]. Either way it is not possible to distinguish whether a species evolved or had pre-adaptations to the conditions in the invaded region using a correlative approach (*i.e.* SDMs) [43]. Thus, we do not know the contribution of these mechanisms to the niche shift observed in India. This species could have filled a niche space absent in its native range but for which it was pre-adapted, or it could have evolved adaptations to the new climate encountered in India. A recent study suggests that many invasive plants have evolved co-adaptations to new environmental conditions in their introduced ranges [44]. Ray *et al.* [45] found that individuals of *L. camara* in India were originated from genetically differentiated native allopatric populations that gradually homogenized. In addition, several ornamental varieties have been produced since its introduction in India through hybridization and artificial selection [29], [46]. Thus, it is possible for mixtures of different genetic pools to have increased the species’ ability to evolve adaptations to novel climates (*i.e.* [47]). However, genetic characterization of populations, reciprocal transplant experiments or a mechanistic modeling approach at a global-scale are required to differentiate between filling a pre-adapted niche and rapid evolution of *L. camara* in India.

The different directions of niche change observed among continents (*i.e.* expansion in India *vs.* contraction in Australia and Africa) may be attributed to contrasting scenarios encountered during the early stages of invasion. Although the first introductions of *L. camara* in these three regions were relatively contemporary (1807–1858), its invasion appeared to have been faster in India [27]. There, *L. camara* extends over 13 million ha whereas in the other two continents it occupies less than 5 million ha [27]. One possible explanation is that the initial introductions in India [27] occurred in highly suitable habitats, as suggested by our model (see Figures 4 b and e), while in Australia and Africa [27] the species arrived to less suitable habitats (see Figures 4 a, c, d and f). A difficult and slow establishment in Australia and Africa could have delayed subsequent invasion phases to leave no sufficient time for *L. camara* to colonize its entire climatic niche, a phenomenon known as the colonization-lag non-equilibrium [48]. Non-equilibrium distributions can be generated by local community or demographic processes that prevent the full occupancy of suitable habitats. For instance, the large extensions of dense forests in Africa and Australia could have acted as a barrier to the dispersal of *L. camara* by inhibiting its growth through light competition [3]. Empirical examination of dispersal capacity, extinction-colonization dynamics and a more precise assessment of the habitat suitability when colonization occurred are necessary to test the non-equilibrium hypothesis. Alternatively, the observed niche contractions could have resulted from genetic bottlenecks during early invasion (*i.e.* [49]–[51]) that reduced the genetic variability of *L. camara,* and its ability to invade its entire niche. Although this is a possible explanation, the number and origin of founders involved in the invasion of Australia and Africa are not known.

Finally, our results highlight the influence that the choice of geographic scale may have on the ability a particular study has to test the hypothesis of niche conservatism. If we had restricted the exotic range to Australia or Africa (*e.g.* [23]) omitting India, we would have missed the evidence that *L. camara* could expand its niche and occupy novel climatic conditions. Biological invasions are a global problem and, thus, a global-scale approach is necessary to test the underlying mechanisms –biogeographic, demographic and evolutionary– involved in this process.

### Niche shift and invasion potential of L. camara

Our capacity to predict long-term changes in the geographic distribution of *L. camara* appears to be hindered by its ability to invade novel environmental conditions. The presence of *L. camara* in warmer climates in India suggests that this plant could invade similar habitats in other regions. In Africa, potentially vulnerable areas include the Democratic Republic of the Congo, Cameroon and Centro-African Republic, where several Reserves and Nationals Parks are located. In Australia, southern Queensland and southeastern Victoria may also be invaded by *L. camara*. While some authors already highlighted the vulnerability of some of these regions [23]–[26], here we add Canberra, Melbourne and their surroundings, and Tasmania to the list of potentially invasible areas (see Figure 5b). In light of possible niche shifts, predictions under climate-change scenarios must be done with caution (*e.g.* [24]).

## Supporting Information

Table S1 **Occurrence records of** ***L. camara*** **used in this study.**
(CSV)

## References

[1] GISIN (2013) Global Invasive Species Information Network, providing free and open access to invasive species data. Available: http://www.gisin.org. Accessed 2013 Dec 18.

[2] Lowe S, Browne M, Boudjelas S, De Poorter M (2000) 100 of the world’s worst invasive alien species: A selection from the global invasive species database. Gland: The Invasive Species Specialist Group (ISSG) a specialist group of the Species Survival Commission (SSC) of the World Conservation Union (IUCN).

[3] Sharma GP, Raghubanshi AS, Singh JS (2005) *Lantana* invasion: An overview. Weed Biol Manag 5: 157–165.

[4] Parsons WT, Cuthbertson EG (2001). Parsons WT, Cuthbertson EG (2001) Noxious weeds of Australia, 2nd edn. Melbourne: CSIRO Publishing.

[5] Johnson S (2008) Review of the declaration of Lantana species in New South Wales. Orange: Department of Primary Industries New South Wales.

[6] Day M, Wiley C, Playford J, Zalucki M (2003) Lantana: Current management, status and future prospects. ACIAR Monograph 102. Canberra: Australian Centre for International Agricultural Research.

[7] Ensbey R (2003) Managing Lantana. NSW Agriculture, Orange. Available: http://www.weeds.org.au/WoNS/lantana/docs/29_NSW_Ag_Fact.pdf. Accessed 2013 Jan 18.

[8] Baars JR, Heystek F (2003) Geographical range and impact of five biocontrol agents established on *Lantana camara* in South Africa. BioControl 48: 743–759.

[9] Ficetola GF, Thuiller W, Miaud C (2007) Prediction and validation of the potential global distribution of a problematic alien invasive species - the American bullfrog. Divers Distrib 13: 476–485.

[10] Muñoz A-R, Real R (2006) Assessing the potential range expansion of the exotic monk parakeet in Spain. Divers Distrib 12: 656–665.

[11] Rouget M, Richardson DM, Nel JL, Le Maitre DC, Egoh B, et al (2004) Mapping the potential ranges of major plant invaders in South Africa, Lesotho and Swaziland using climatic suitability. Divers Distrib 10: 475–484.

[12] Colwell RK, Rangel TF (2009) Colloquium Papers: Hutchinson's duality: The once and future niche. Proc Natl Acad Sci 106: 19651–19658. 19805163 10.1073/pnas.0901650106 PMC2780946

[13] Elith J, Leathwick JR (2009) Species Distribution Models: Ecological explanation and prediction across space and time. Annu Rev Ecol Evol Syst 40: 677–697.

[14] Guisan A, Thuiller W (2005) Predicting species distribution: offering more than simple habitat models. Ecol Lett 8: 993–1009. 10.1111/j.1461-0248.2005.00792.x 34517687

[15] Holt R, Gaines M (1992) Analysis of adaptation in heterogeneous landscapes: Implications for the evolution of fundamental niches. Evol Ecol 6: 433–447.

[16] Peterson AT, Soberón J, Sanchez-Cordero V (1999) Conservatism of ecological niches in evolutionary time. Science 285: 1265–1267. 10455053 10.1126/science.285.5431.1265

[17] Prinzing A (2001) The niche of higher plants: evidence for phylogenetic conservatism. Proc R Soc Lond B Biol Sci 268: 2383–2389. 10.1098/rspb.2001.1801 PMC1088890 11703879

[18] Wiens JJ, Ackerly DD, Allen AP, Anacker BL, Buckley LB, et al (2010) Niche conservatism as an emerging principle in ecology and conservation biology. Ecol Lett 13: 1310–1324. 20649638 10.1111/j.1461-0248.2010.01515.x

[19] Broennimann O, Treier UA, Müller-Schärer H, Thuiller W, Peterson AT, et al (2007) Evidence of climatic niche shift during biological invasion. Ecol Lett 10: 701–709. 17594425 10.1111/j.1461-0248.2007.01060.x

[20] Gallagher RV, Beaumont LJ, Hughes L, Leishman MR (2010) Evidence for climatic niche and biome shifts between native and novel ranges in plant species introduced to Australia. J Ecol 98: 790–799.

[21] Petitpierre B, Kueffer C, Broennimann O, Randin C, Daehler C, et al (2012) Climatic niche shifts are rare among terrestrial plant invaders. Science 335: 1344–1348. 22422981 10.1126/science.1215933

[22] Gallien L, Douzet R, Pratte S, Zimmermann NE, Thuiller W (2012) Invasive species distribution models – how violating the equilibrium assumption can create new insights. Glob Ecol Biogeogr 21: 1126–1136.

[23] Taylor S, Kumar L, Reid N (2012) Impacts of climate change and land-use on the potential distribution of an invasive weed: a case study of Lantana camara in Australia. Weed Res 52: 391–401.

[24] Taylor S, Kumar L, Reid N, Kriticos DJ (2012) Climate Change and the Potential Distribution of an Invasive Shrub, *Lantana camara* L. PLoS. One7: e35565. 10.1371/journal.pone.0035565 PMC3334920 22536408

[25] Van Oosterhout E, Clark A, Day MD, Menzies E (2004) *Lantana* control manual: Current management and control options for *Lantana* (*Lantana camara*) in Australian State of Queensland. Department of Natural Resources, Mines and Enegry, Brisbane, Australia. Available: http://www.nrm.qld.gov.au/pests/wons/Lantana. Accessed 2013 Jan 18.

[26] Lüi XR (2011) Quantitative risk analysis and prediction of potential distribution areas of common lantana (*Lantana camara*) in China. Comput Ecol and Softw 2: 60–65.

[27] Bhagwat SA, Breman E, Thekaekara T, Thornton TF, Willis KJ (2012) A battle lost? Report on two centuries of invasion and management of *Lantana camara* L. in Australia, India and South Africa. PLoS One7: e32407. 10.1371/journal.pone.0032407 PMC3293794 22403653

[28] Sanders RW (1987) Taxonomic significance of chromosome observations in Caribbean species of *Lantana* (Verbenaceae). Am J Bot 74: 914–920.

[29] Sanders RW (2006) Taxonomy of *Lantana* sect. *Lantana* (Verbenaceae): I. correct application of *Lantana camara* and associated names. SIDA 22: 381–421.

[30] Smith LS, Smith DA (1982) The naturalised *Lantana camara* complex in eastern Australia. Queensland Bot Bull 1: 1–26.

[31] Broennimann O, Fitzpatrick MC, Pearman PB, Petitpierre B, Pellissier L, et al (2012) Measuring ecological niche overlap from occurrence and spatial environmental data. Glob Ecol Biogeogr 21: 481–497.

[32] Howard RA (1969) A check list of cultivar names used in the genus *Lantana* . Arnoldia 29: 73–109.

[33] Swarbrick JT, Willson BW, Hannan-Jones MA (1995) The biology of australian weeds 25. *Lantana camara* L. Plant Prot Q 10: 82–82.

[34] Hijmans RJ, Cameron SE, Parra JL, Jones PG, Jarvis A (2005) Very high resolution interpolated climate surfaces for global land areas. Int J Climatol 25: 1965–1978.

[35] R-Development-Core-Team (2010) R: A language and environment for statistical computing, Vienna, Austria.

[36] Warren DL, Glor RE, Turelli M (2008) Environmental niche equivalency versus conservatism: quantitative approaches to niche evolution. Evolution 62: 2868–2883. 18752605 10.1111/j.1558-5646.2008.00482.x

[37] Bradley BA (2012) Distribution models of invasive plants over-estimate potential impact. Biol Invasions 15: 1417–1429.

[38] Phillips SJ, Anderson RP, Schapire RE (2006) Maximum entropy modeling of species geographic distributions. Ecol Model 190: 231–259.

[39] Peterson A, Soberón J, Pearson RG, Anderson R, Martinez-Meyer E, et al.. (2011) Ecological niches and geographic distributions. Oxford: Princeton University Press.

[40] Elith J, Kearney M, Phillips S (2010) The art of modelling range-shifting species. Methods Ecol Evol 1: 330–342.

[41] Webber BL, Le Maitre DC, Kriticos DJ (2012) Comment on “Climatic niche shifts are rare among terrestrial plant invaders”. Science 338: 193–193. 10.1126/science.1225980 23066061

[42] Mukherjee A, Williams DA, Wheeler GS, Cuda JP, Pal S, et al (2011) Brazilian peppertree (*Schinus terebinthifolius*) in Florida and South America: evidence of a possible niche shift driven by hybridization. Biol Invasions 14: 1415–1430.

[43] Tingley R, Vallinoto M, Sequeira F, Kearney MR (2014) Realized niche shift during a global biological invasion. PNAS 111: 10233–10238. 24982155 10.1073/pnas.1405766111 PMC4104887

[44] Maron JL, Vila M, Bommarco R, Elmendorf S, Beardsley P (2004) Rapid evolution of an invasive plant. Ecol Monogr 74: 261–280.

[45] Ray A, Quader S, Loudet O (2013) Genetic diversity and population structure of “*Lantana camara”* in India indicates multiple introductions and gene flow. Plant Biol doi:10.1111/plb.12087. 10.1111/plb.12087 24119091

[46] Stirton CH (1977) Some thoughts on the polyploid *Lantana camara* L, (Verbenaccae). In Proceedings of the Second National Weeds Conference, Stellenbosch, South Africa. Cape Town: Balkema. 321–340.

[47] Ellstrand N, Schierenbeck K (2000) Hybridization as a stimulus for the evolution of invasiveness in plants? Proc Natl Acad Sci 97: 7043–7050. 10860969 10.1073/pnas.97.13.7043 PMC34382

[48] De Marco P, Diniz JAF, Bini LM (2008) Spatial analysis improves species distribution modelling during range expansion. Biol Lett 4: 577–580. 18664417 10.1098/rsbl.2008.0210 PMC2610070

[49] Baker AJ, Moeed A (1987) Rapid genetic differentiation and founder effect in colonizing populations of common mynas (*Acridotheres tristis*). Evolution: 525–538. 10.1111/j.1558-5646.1987.tb05823.x 28563814

[50] Dlugosch KM, Parker IM (2008) Founding events in species invasions: genetic variation, adaptive evolution, and the role of multiple introductions. Mol Ecol 17: 431–449. 17908213 10.1111/j.1365-294X.2007.03538.x

[51] Easteal S (1989) The effects of genetic drift during range expansion on geographical patterns of variation: a computer simulation of the colonization of Australia by *Bufo marinus* . Biol J Linn Soc Lond 37: 281–295.

